# Formulation Development of Directly Compressible Tablets Incorporating Trisamo Extract With Synergistic Antioxidant Activity

**DOI:** 10.1155/2024/8920060

**Published:** 2024-10-09

**Authors:** Jirapornchai Suksaeree, Thaniya Wunnakup, Natawat Chankana, Laksana Charoenchai, Chaowalit Monton

**Affiliations:** ^1^Department of Pharmaceutical Chemistry, College of Pharmacy, Rangsit University, Pathum Thani 12000, Thailand; ^2^Drug and Herbal Product Research and Development Center, College of Pharmacy, Rangsit University, Pathum Thani 12000, Thailand; ^3^Sun Herb Thai Chinese Manufacturing, College of Pharmacy, Rangsit University, Pathum Thani 12000, Thailand

**Keywords:** Box–Behnken design, combination index, design space, optimization, simplex lattice design

## Abstract

This work investigates the synergistic antioxidant activity of the compositions of Trisamo (TSM) herbal formula containing the dried fruits of *Terminalia chebula*, *Terminalia arjuna*, and *Terminalia bellirica*. An augmented simplex lattice design was utilized to investigate the synergistic antioxidant activity, finding an equal mass ratio among the three herbal drugs to exhibit optimal synergistic antioxidant activity, with a combination index of less than 0.8. The optimal TSM extract was used to prepare directly compressible tablets employing a Box–Behnken design response surface methodology, optimizing compressional force (500, 1000, and 1500 psi), sodium starch glycolate (0%, 2%, and 4%), and magnesium stearate (0.5%, 1.0%, and 1.5%). Optimal parameters were a compressional force of 1000 psi, 2% sodium starch glycolate, and 0.5% magnesium stearate. The TSM extract tablet had a weight of 600.06 mg, a diameter of 12.78 mm, a thickness of 4.12 mm, a hardness of 6.85 kP, a friability of 0.30%, and a disintegration time of 1.81 min. Computer model predictions were verified with a low percentage error (≤ 10.00%). After 6 h, phenolic compounds were dissolved to an extent of approximately 40%–80%, including gallic acid (57.11%), corilagin (38.64%), chebulagic acid (58.49%), and chebulinic acid (81.44%). Stability data revealed that the phenolic compounds were retained for 3 months compared to the initial time point, with gallic acid at 81.43% and 100.27%, corilagin at 94.81% and 87.85%, chebulagic acid at 92.22% and 69.83%, and chebulinic acid at 107.00% and 85.54% at 30°C/75% RH and 45°C/75% RH, respectively. The summation of these four compounds did not change significantly when stored under either set of conditions. In summary, mixture design and response surface design were successfully utilized in the optimization of TSM extract tablets with synergistic antioxidant activity.

## 1. Introduction

Trisamo (TSM) is a traditional Thai herbal remedy composed of the dried fruits of the three *Terminalia* plants: *Terminalia chebula* (TC), *Terminalia arjuna* (TA), and *Terminalia bellirica* (TB) in an equal mass ratio. TSM has a long history of use in Thai traditional medicine to treat fever and cough, enhance overall health, and alleviate abdominal bloating [1]. In animal studies, a water extract of TSM has demonstrated antipyretic and analgesic properties. Its antipyretic effects are attributed to the inhibition of prostaglandins, while its analgesic effects potentially involve the suppression of pain mediator synthesis or release [1]. The ethanolic extract of TSM exhibits anti-*Escherichia coli* and *Salmonella* spp. properties [2]. Moreover, the *Terminalia* plants contain various phenolic compounds with well-known antioxidant properties, which likely contribute to TSM's substantial antioxidant activity [3].

Combinations of herbal drugs can amplify their therapeutic effects or biological activities by utilizing advantageous interactions between their diverse constituents [4]. Numerous studies have documented the presence of synergistic effects among different medicinal plants, with traditional Chinese medicines often showcasing such synergy [5–10]. It is common practice to combine multiple herbal drugs to enhance therapeutic efficacy and reduce potential side effects. Synergism can occur between the herbs and other components within a prescription, as well as between the active constituents of the herbs [11, 12].

The importance of synergistic antioxidant activity in compressed tablets lies in its potential to enhance the therapeutic efficacy and stability of the formulation. Synergistic antioxidants, when combined, can exhibit greater overall antioxidant capacity than individual components alone, providing more effective protection against oxidative stress. This is crucial in developing tablets with improved health benefits, as oxidative stress is linked to various chronic diseases and aging processes [13–17].

Incorporating synergistic antioxidants into compressed tablets can lead to several key advantages, including improved stability (tablets with optimized antioxidant formulations can have better shelf stability, reducing the risk of degradation over time and maintaining their therapeutic effectiveness) [18, 19], reduced dosage requirements (the increased potency of synergistic antioxidants may allow for lower dosages to achieve the same or greater therapeutic effect, potentially minimizing side effects and improving patient compliance) [20, 21], and commercial viability (tablets that effectively combine synergistic antioxidants can be more attractive in the market, offering added value to consumers seeking effective health supplements with proven benefits) [22]. Overall, integrating synergistic antioxidants into compressed tablets represents a significant advancement in pharmaceutical formulation, enhancing both the efficacy and practical benefits of health supplements.

Traditional experimentation often relies on the trial-and-error method, which can be inefficient and lack structure, particularly without expertise in the subject matter. Additionally, the one factor at a time (OFAT) approach is utilized, where one parameter is altered before measuring the response and repeating the process with another. However, this method rarely identifies the optimal set of conditions involving multiple factors [23]. Design of experiments (DOEs) addresses these limitations, offering several advantages. It saves time, budget, and resources by employing a small number of tests, uncovers interactions between factors, and characterizes response surfaces [24–26]. Furthermore, a statistical model can be employed to predict the impact of multiple factors simultaneously [23].

Previously, the authors successfully utilized a simplex lattice design to detect the level of synergistic antioxidant activity for herbal drug compositions contained in the Chatuphalathika herbal formula (a mixture of TC, TA, TB, and *Phyllanthus emblica* in equal mass ratio) based on the combination index (CI) approach [27]. However, other herbal formulas like Triphala and TSM with similar herbal compositions did not prove their synergistic antioxidant activity. However, the previous works did prove the positive interaction of the bioactive constituents of TSM, Triphala, and Chatuphalathika, particularly in terms of the phenolic compounds which are increased with respect to the single herbal components [3, 28, 29]. However, the synergistic effect of the composition of the TSM formula has not yet been proven. Therefore, this work is aimed at evaluating the synergistic antioxidant activity of varying compositions of the TSM formula using an experimental design called simplex lattice design. Furthermore, the extract of the three drugs in a ratio giving optimal synergistic antioxidant activity was used to prepare directly compressible tablets using a response surface methodology known as the Box–Behnken design.

This research offers a novel evaluation of the synergistic effects of TSM. While TSM has historical use in Thai traditional medicine, its synergistic antioxidant activity was previously unexplored. By employing advanced experimental designs, including simplex lattice and Box–Behnken designs, this study systematically investigates the interaction effects among TSM herbal constituents. This approach provides a structured, data-driven analysis of TSM's synergistic potential and practical application, including the development of directly compressible tablets. These results represent a significant advancement beyond traditional experimental methods to offer a precise evaluation of herbal efficacy.

## 2. Materials and Methods

### 2.1. Materials

Four standard compounds, including gallic acid, corilagin, chebulagic acid, and chebulinic acid, were obtained from Chengdu Biopurify Phytochemicals Ltd., Sichuan, China. The source of the dried fruits of TC, TA, and TB for this study was Charoensuk Osod, Nakhon Pathom, Thailand. All pharmaceutical additives were pharmaceutical grade. Analytical grade reagents and HPLC grade solvents were employed.

### 2.2. Synergistic Antioxidant Activity Evaluation Using Augmented Simplex Lattice Design

The mass ratio modeling for the mixture of dried fruit powders of TC, TA, and TB was executed using an augmented simplex lattice design. This design incorporated six simplex points, three axial points, and a center point, with the center point being independently tested in triplicate (as detailed in Table 1). A total of 6 g of the herbal drug powder mixture, with specific mass ratios indicated in Table 1, was placed into a tea bag and sealed. The tea bag was then boiled in a 100-mL beaker in 40 mL of water for 15 min. This extraction process was repeated three times, with the filtrate collected each time using Whatman filter paper (No. 1) by vacuum filtration. The three filtrates were combined, cooled, frozen, and lyophilized with a freeze-dryer [3]. The resulting lyophilized extract was analyzed for three responses: total phenolic content (TPC), half-maximal inhibitory concentration (IC_50_) by the DPPH assay, and IC_50_ by the ferric reducing antioxidant power (FRAP) assay as described in the previous study [30].

To study the synergistic antioxidant activity of the TSM extracts, the response additivity approach was employed [31]. The CI was utilized as a tool to assess the synergistic effect, calculated using Equation (1).

(1) $$\begin{array}{c} \text{CI}=\frac{{\text{IC}}_{50\;\left(\text{Combination}\right)}}{f_{A}{\text{IC}}_{50\;\left(A\right)}+f_{B}{\text{IC}}_{50\;\left(B\right)}+f_{C}{\text{IC}}_{50\;\left(C\right)}} \end{array}$$

where IC_50 (Combination)_ refers to the IC_50_ of the extract of the combination; *f*_*A*_, *f*_*B*_, and *f*_*C*_ represent the mass ratios or mass fractions of TC, TA, and TB, respectively; and IC_50(*A*)_, IC_50(*B*)_, and IC_50(*C*)_ denote the IC_50_ of the individual extracts of TC, TA, and TB, respectively. CI values less than 1, equal to 1, and greater than 1 were considered indicative of synergistic, additive, and antagonistic effects, respectively [32]. The design space was defined where the CI value was equal to or less than 0.8.

### 2.3. Preparation and Optimization of Directly Compressible Tablets

The Box–Behnken design was employed to optimize the formulation and preparation method for the TSM extract tablets. Levels for each factor were selected based on preliminary studies and the authors' experience. Seventeen formulations were tested, consisting of twelve unique formulations and five replicated formulations. The formulations varied in three factors: compressional force, quantity of sodium starch glycolate (SSG), and quantity of magnesium stearate (MgSt), as indicated in Table 2.

The directly compressible TSM extract tablets were composed of several ingredients. The optimal TSM extract powder was combined with SSG (a disintegrant), colloidal silicon dioxide (a glidant), MgSt (a lubricant), talcum (an antiadherent), and microcrystalline cellulose PH102 (a diluent and dry binder). All the ingredients were passed through a 60-mesh sieve. In a 100-g formulation, 33.3 g of TSM extract powder was first blended with half of the microcrystalline cellulose. The remaining half of the microcrystalline cellulose was gently mixed for 1 min with SSG (in amounts ranging from 0 to 4 g), talc (5 g), colloidal silicon dioxide (1 g), and MgSt (0.5% to 1.5%). The two mixtures were subsequently combined and mixed thoroughly for 3 min to ensure homogeneity. The resulting powder mixture was then weighed out in 600 mg portions, each containing 200 mg of TSM extract, and was compressed into tablets using an in-house assembled hydraulic press. This press was connected to a pressure gauge and equipped with a die of 12.8 mm internal diameter, applying specific compressional forces as outlined in Table 2.

The tablets were assessed for various physical properties, including weight and weight variation, diameter and thickness, hardness, friability, and disintegration time (DT). To generate a contour plot using Design-Expert v11, only thickness, hardness, friability, and DT were considered. The diameter was not included in the construction of the contour plot because it was fixed by the die size. Additionally, weight and weight variation did not produce a contour plot as each tablet was weighed individually, ensuring consistency. Analysis of variance (ANOVA) data and perturbation plots were provided, and design spaces were established. These design spaces were characterized by a hardness range of 5–9 kP, friability of less than 1%, and a DT of less than 15 min. Formulations within these parameters were chosen for the verification step. The optimal formulation was then prepared, and its physical properties were evaluated. Additionally, the percentage error of the predictions was reported.

### 2.4. Tablet Physical Property Evaluation

An analytical balance (Entris224i-1S, Sartorius AG, Göttingen, Germany) was used to evaluate the weight and weight variation of 20 tablets containing TSM extract. A digital thickness gauge was used to measure the diameter and thickness of 20 TSM extract tablets. The hardness of 10 TSM extract tablets was assessed using a Stokes–Monsanto hardness tester. The friability of 11 tablets was evaluated using a friability tester (Model: CS-2, Tianjin Guoming Medicinal Equipment Co. Ltd., Tianjin, China). A disintegration tester (Model: BJ-2, Tianjin Guoming Medicinal Equipment Co. Ltd., Tianjin, China) was used to evaluate the DT of six tablets in water at 37 ± 0.5°C.

### 2.5. Phenolic Constituent Content Analysis

To quantify four phenolic compounds (gallic acid, corilagin, chebulagic acid, and chebulinic acid), a validated HPLC analysis method similar to that reported in a previously conducted study [3] was employed.

### 2.6. Dissolution Test

Three optimized TSM extract tablets underwent dissolution testing using dissolution apparatus II (Model: 72-600-400, Hanson Research Corp., California, United States). The paddle speed was set at 100 ± 1 rpm, and the dissolution medium consisted of a 0.5% sodium lauryl sulfate solution (900 mL) maintained at 37 ± 0.5°C. Samples of 5 mL were withdrawn at specified intervals over 6 h, with fresh medium added to maintain a constant volume. The collected samples were filtered and analyzed by HPLC to construct dissolution profiles for each phenolic compound.

### 2.7. Stability Test

The optimized TSM extract tablets were carefully placed in a polyethylene terephthalate (PET) bottle with a desiccant and sealed using a pressure-sensitive cap with a foam liner. The bottle was then capped with an aluminum-coated plastic cap. These bottled tablets underwent stability testing in a climate chamber (Memmert GmbH+Co. KG, Schwabach, Germany) under two temperature and humidity conditions (30 ± 2°C/75 ± 5% RH and 45 ± 2°C/75 ± 5% RH) for 3 months. Throughout this period, the levels of phenolic compounds were monitored and compared with the initial measurements.

### 2.8. Statistical Analysis

To assess differences among multiple groups, statistical analysis utilized a one-way ANOVA followed by Tukey's honestly significant difference (HSD) post hoc test. Statistical calculations were conducted using SPSS Statistics 22.0 (IBM, New York, United States). Statistical significance was set at *p* < 0.05 with a 95% confidence interval considered as indicative of significance.

## 3. Results

### 3.1. Synergistic Antioxidant Activity TSM Herbal Formula

**Figure 1:**
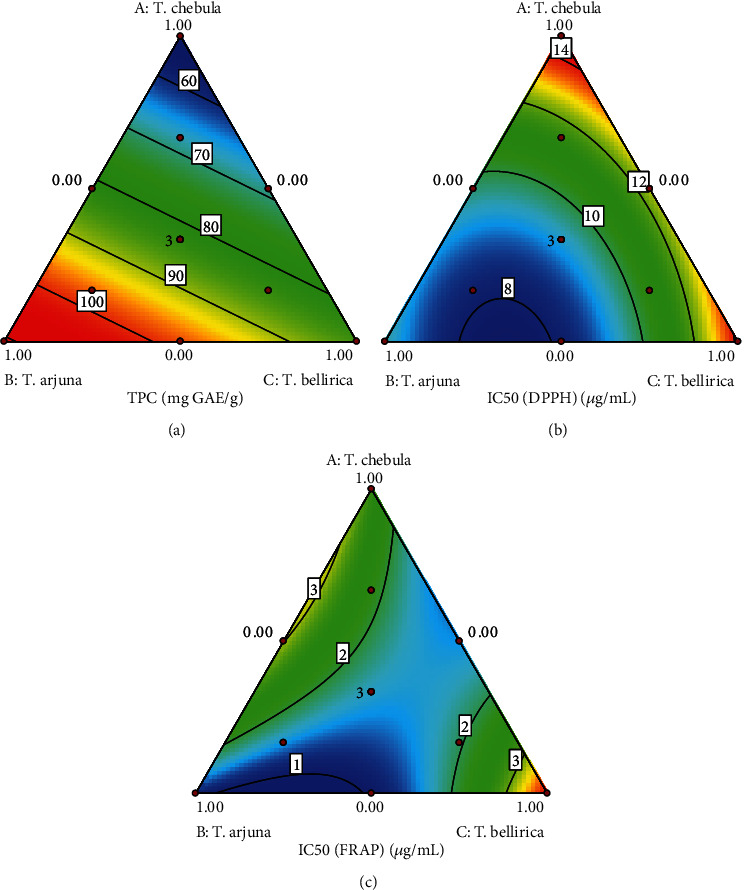
Contour plots of (a) TPC, (b) IC_50_ values obtained from DPPH assay, and (c) IC_50_ values obtained from FRAP assay.

The synergistic antioxidant activity of the herbal drug composition in the TSM formula was evaluated using an augmented simplex lattice design. Firstly, contour plots were generated from the TPC and antioxidant activity in terms of IC_50_ values obtained from DPPH and FRAP assays. The plots revealed that increasing the mass ratio of TC decreased TPC. Increasing TA increased TPC, while increasing TB did not affect TPC (Figure 1(a)). In the case of antioxidant activity based on IC_50_ from the DPPH assay, decreasing the mass ratios of TC or TB increased antioxidant activity in terms of decreasing IC_50_ value, while increasing TA increased antioxidant activity (Figure 1(b)). In the case of the FRAP assay, reducing the mass ratio of TC decreased IC_50_ and increased antioxidant activity, while increasing TA had no effect. For TB, the lowest IC_50_ value was achieved at the medium mass ratio (Figure 1(c)).

**Figure 2:**
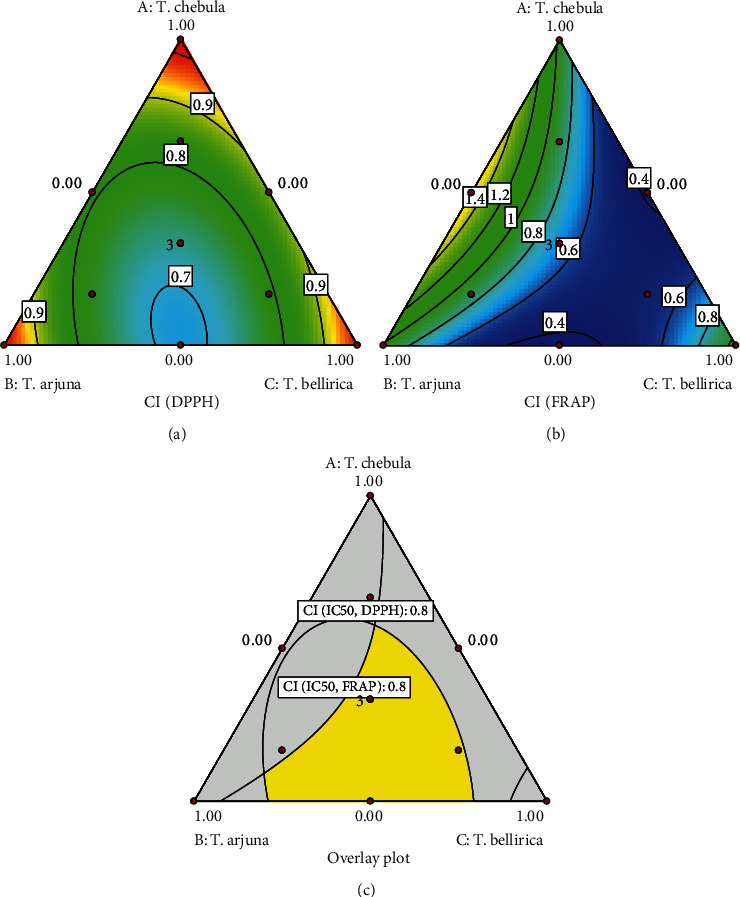
Contour plots of CI values of (a) IC_50_ values obtained from DPPH assay, (b) IC_50_ values obtained from FRAP assay, and (c) overlay plots of the CI of IC_50_ values obtained from DPPH and FRAP assays, where the value was equal to or lower than 0.8.

Synergistic antioxidant activity was evaluated using the CI method. Only CI values of IC_50_ from DPPH and FRAP assays were used for analysis (Figure 2). The design space where the CI values were simultaneously equal to or less than 0.8 for IC_50_ from both DPPH and FRAP assays is shown in Figure 2(c). The results indicate that the original TSM formula comprising an equal mass ratio of the three drugs exhibited synergistic antioxidant activity. Verification data are presented in Table 3, demonstrating that the CI values of the IC_50_ values obtained from DPPH and FRAP assays were both close to the predicted values, with a percentage error of less than 10.00%. This confirms that the predictions made by the Design-Expert were accurate. Therefore, the TSM extract obtained from this optimal mass ratio was used as the active ingredient for the preparation of directly compressible tablets.

### 3.2. Optimal Tablet Formulation and Preparation Conditions and Tablet Properties

The TSM extract with optimal synergistic antioxidant activity was further used to prepare the tablets by the direct compression method. The physical properties of the tablets were evaluated in terms of the compressional forces, the quantity of SSG, and the quantity of MgSt applied in their preparation.

**Figure 3:**
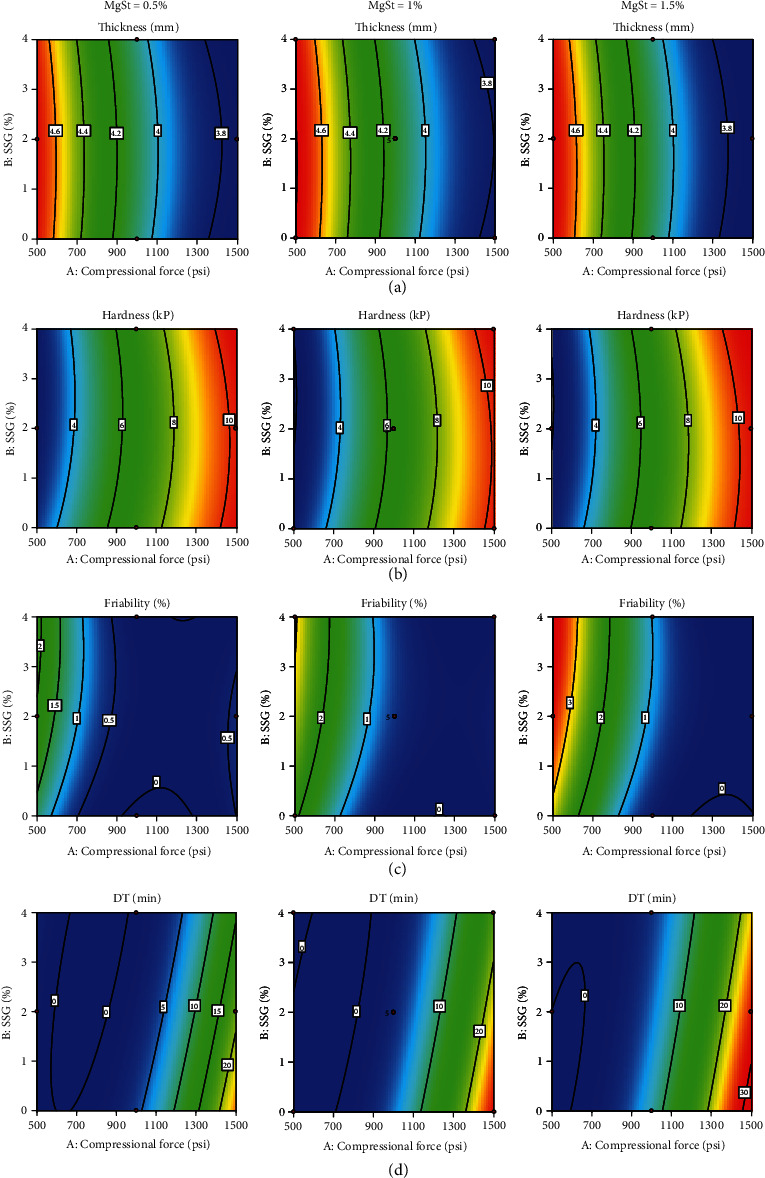
Contour plots of (a) thickness, (b) hardness, (c) friability, and (d) DT of directly compressible tablets incorporating TSM extract with synergistic antioxidant activity when different levels of MgSt were used: 0.5% (left), 1% (middle), and 1.5% (right).

**Figure 4:**
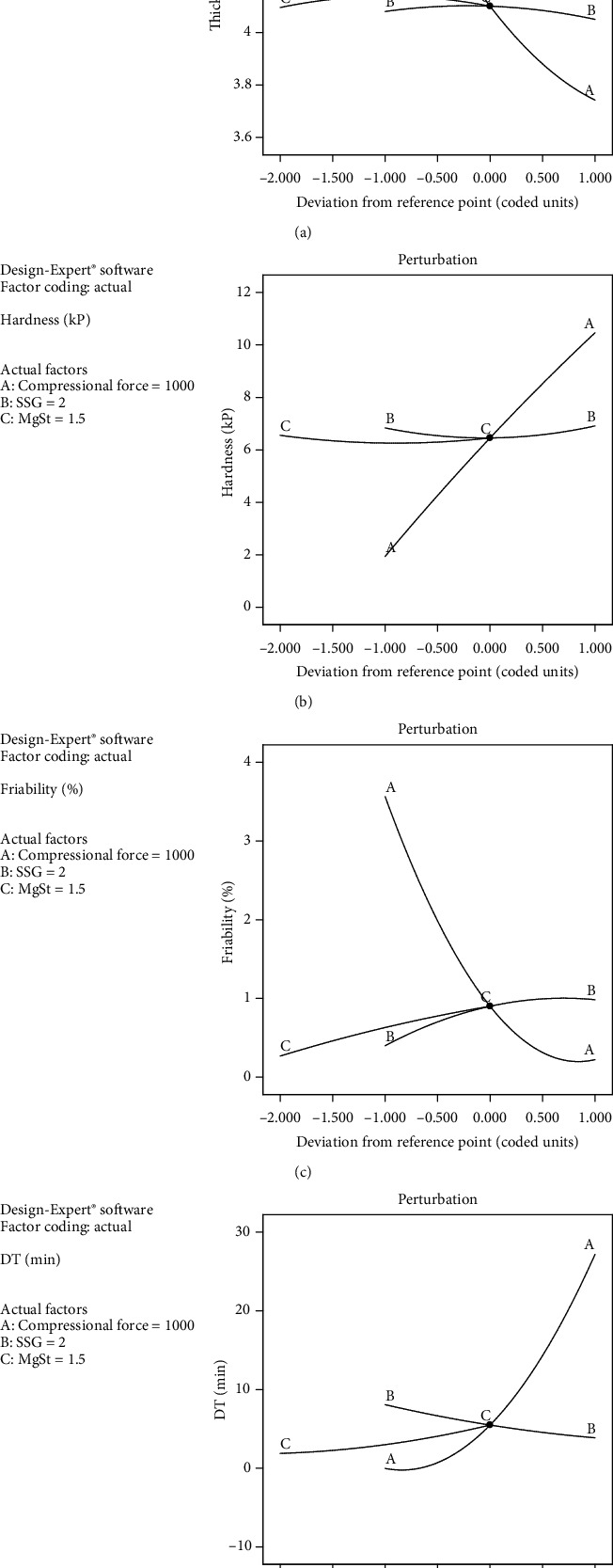
Perturbation plots of (a) thickness, (b) hardness, (c) friability, and (d) DT of directly compressible tablets incorporating TSM extract with synergistic antioxidant activity. A, B, and C denote compressional force, the quantity of SSG, and the quantity of MgSt, respectively.

Contour plots and perturbation plots of the tablet's physical properties are shown in Figures 3 and 4, respectively. Figures 3 and 4 and Table 4 reveal that increasing compressional force significantly decreased tablet thickness and friability but significantly increased hardness and significantly prolonged DT. An increasing quantity of SSG did not affect thickness and hardness but insignificantly increased friability and significantly shortened DT. Increasing the quantity of MgSt did not affect thickness and hardness but significantly increased friability and significantly prolonged DT.

In terms of factor interactions, the interaction between compressional force and quantity of SSG significantly increased hardness but significantly shortened DT. Interaction between compressional force and quantity of MgSt significantly decreased friability and significantly prolonged DT. Interaction between quantities of SSG and MgSt did not show any significant effect on the physical properties of the tablets. In the case of quadratic terms of compressional force, it significantly increased thickness, friability, and DT but significantly decreased hardness. When quadratic terms of quantity of SSG or MgSt were increased, thickness significantly decreased while hardness significantly increased. The lack of fit for all responses was insignificant, indicating that the model was fitted and can be used to predict the responses.

**Figure 5:**
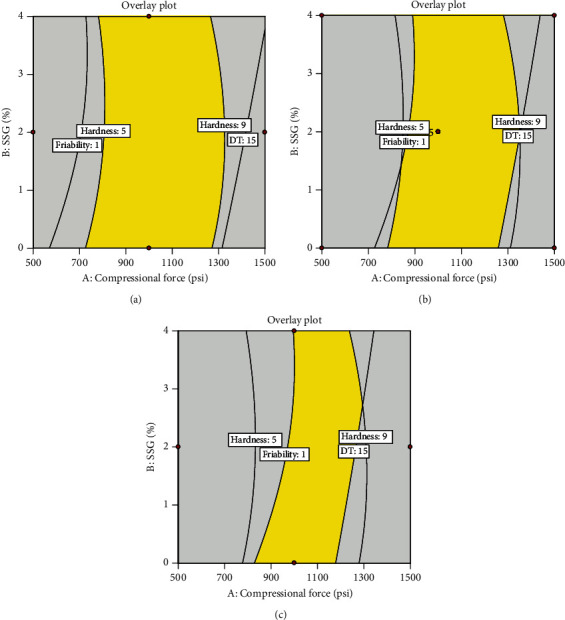
Design spaces for hardness of 5–9 kP, friability of not more than 1%, and DT of not more than 15 min when different levels of MgSt were used: (a) 0.5%, (b) 1%, and (c) 1.5%.

Design spaces where hardness of 5–9 kP, friability of not more than 1%, and DT of not more than 15 min were constructed as shown in Figure 5. A compressional force of 1000 psi was the optimum force, while compressional forces of 500 and 1500 psi were outside the design space. Increasing the quantity of MgSt decreased the area of design space, so 0.5% MgSt was selected as it provided a larger area of design space than 1% or 1.5%. A quantity of SSG of 2% was selected since it was located at the center of the design space. Therefore, the optimal condition of the compressional force of 1000 psi, SSG of 2%, and MgSt of 0.5% was further investigated in the verification step. This optimal tablet formulation was composed of 33.3% TSM extract, 2% SSG, 0.5% MgSt, 5% talcum, 1% colloidal silicon dioxide, and 58.2% microcrystalline cellulose, which was equivalent to 200, 12, 3, 30, 6, and 349.2 mg of the ingredients per tablet, respectively. Verification data are shown in Table 5. The actual values were close to the predicted values. The percentage error of friability was 10%, while the error in the other responses was less than 5%, indicating that the prediction by the Design-Expert program was accurate and reliable.

**Figure 6:**
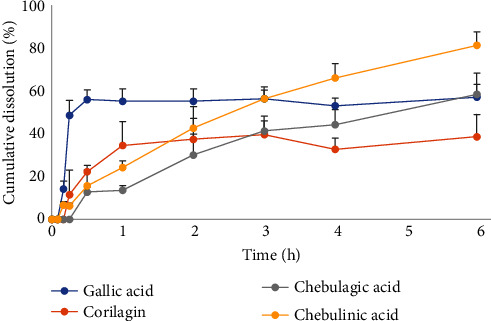
Dissolution profiles of phenolic compounds contained in directly compressible tablets incorporating TSM extract with synergistic antioxidant activity using 0.5% sodium lauryl sulfate solution as the dissolution medium.

The optimal tablet formulation was further evaluated for the dissolution of specific phenolic compounds in a 0.5% sodium lauryl sulfate solution. The dissolution profiles of gallic acid and corilagin appeared constant within 30 min and 2 h, respectively. Chebulagic acid and chebulinic acid increased up to 6 h. The maximum dissolution of corilagin, gallic acid, chebulagic acid, and chebulinic acid within 6 h were 38.65 ± 10.41%, 57.11 ± 6.06%, 58.49 ± 9.88%, and 81.44 ± 6.30%, respectively (Figure 6).

**Figure 7:**
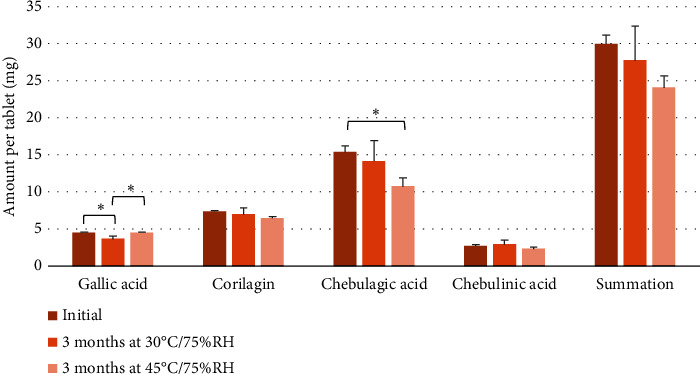
Stability of phenolic compounds contained in directly compressible tablets incorporating TSM extract with synergistic antioxidant activity when stored at 30 ± 2°C/75 ± 5% RH and 45 ± 2°C/75 ± 5% RH for 3 months. An asterisk (^∗^) denotes significant values (*p* < 0.05) obtained from one-way ANOVA followed by Tukey's HSD post hoc analysis.

The stability of the optimized tablet formulation was also assessed under conditions of 30°C/75% RH and 45°C/75% RH for 3 months. Initially, the tablets contained 4.51 ± 0.07 mg of gallic acid, 7.36 ± 0.14 mg of corilagin, 15.38 ± 0.84 mg of chebulagic acid, and 2.73 ± 0.18 mg of chebulinic acid. The contents of corilagin and chebulinic acid remained unchanged throughout the storage period. The gallic acid content in tablets stored at 30°C/75% RH decreased significantly compared to the initial measurement, while no change was observed in tablets stored at 45°C/75% RH. The chebulagic acid content in tablets stored at 30°C/75% RH remained stable, whereas a significant decrease was noted in tablets stored at 45°C/75% RH compared to the initial level. The total content of all four phenolic compounds did not change significantly when stored at either 30°C/75% RH or 45°C/75% RH for 3 months (Figure 7). The percentage of remaining phenolic compounds at 30°C/75% RH and 45°C/75% RH, respectively, was as follows: gallic acid 81.43% and 100.27%, corilagin 94.81% and 87.85%, chebulagic acid 92.22% and 69.83%, and chebulinic acid 107.00% and 85.54%. The summation of these four compounds was 92.58% and 80.26% when stored at 30°C/75% RH and 45°C/75% RH, respectively.

## 4. Discussion

Formerly, CI was used to determine the level of the positive interactions among plant constituents in the herbal formulae of Triphala [29], TSM [3], and Chatuphalathika [28]. Later, it was successfully applied to determine the synergistic antioxidant activity of three plants used in herbal beverages [33] and the Chatuphalathika formula [27]. To the best of our knowledge, this is the first report on the synergistic antioxidant activity of the TSM formula. The original formula of TSM comprises an equal mass ratio of three *Terminalia* fruits and exhibits optimal synergistic antioxidant activity. This evidence supports the wisdom of traditional medicine that can combine herbal plants to promote activity superior to that of single herbal drugs.

This research investigated various factors involved in the formulation of tablets, namely, compressional force, the quantity of SSG, and the quantity of MgSt. Typically, increasing the compressional force resulted in a decrease in tablet thickness and friability but an increase in tablet hardness and DT. The findings of this study are consistent with previous research [30, 34–40]. Previous work reported that increasing compressional force did not impact the DT when SSG was used [41]. However, this study revealed that the interaction between compressional force and the quantity of SSG significantly increased hardness while significantly reducing DT.

SSG was chosen as a disintegrant due to its ability to shorten the DT of the drug formulation through a swelling mechanism. When the tablet comes into contact with an aqueous medium, the disintegrant rapidly absorbs water, causing it to swell and generate pressure. This pressure pushes the particles apart, leading to the disintegration of the tablet [42, 43]. The effectiveness of many disintegrants is influenced by the presence of hydrophobic ingredients like lubricants. However, SSG is not affected by certain hydrophobic ingredients [41, 44]. Based on the ANOVA results, the present study found that SSG did not interact with MgSt, which is a hydrophobic substance. However, the quadratic terms of SSG had a significant impact on tablet thickness and hardness, while showing no effect on friability and DT. SSG is not typically used in directly compressible tablets due to its natural tendency to rapidly disintegrate. However, the authors discovered that increasing the compressional force to enhance hardness and reduce tablet friability led to a significant prolongation of the DT of the TSM extract tablets developed in this work. Therefore, the authors incorporated SSG to shorten the DT of specific tablet formulations where high compressional force was applied. Furthermore, the high sugar content in the fruits of the herbal drugs comprising the TSM formula may cause difficulty in disintegration [45]. MgSt functions as a lubricant, helping to reduce resistance between particles. Its hydrophobic nature allows for an extended DT [46], as also found in this research.

Typically, the duration of the dissolution test for immediate-release dosage forms is 1–2 h. However, this research tested for up to 6 h to assess the dissolution pattern of the phenolic compounds of TSM. The dissolution profiles of gallic acid and corilagin were similar, reaching the plateau phase within 2 h. The dissolution profiles of chebulagic acid and chebulinic acid were also similar, increasing throughout the 6-h test. According to the compound polarity, gallic acid and corilagin are more polar than chebulagic acid and chebulinic acid [47]. Hence, the pairs of compounds could dissolve with similar profiles. The more polar compound gallic acid could be dissolved in a polar solvent like 0.5% sodium lauryl sulfate solution. However, this study found that dissolution did not approach 100% after 6 h. It can be described by the hygroscopic property of the TSM extract due to it was dried by freeze drying. Furthermore, the herbal drugs comprising the TSM formula are fruits with high sugar content [45] that could produce a sticky mass before or during the dissolution test. The standard markers may not dissolve freely in the medium, resulting in their lower dissolution measurements.

Although the stability data suggested no significant changes when stored at 30°C/75% RH or 45°C/75% RH for 3 months, a test of longer duration should be carried out to verify long-term stability. Guidelines recommend conducting the test at 30 ± 2°C/75 ± 5% RH with a duration of 24 months and annually through the proposed shelf life [48].

## 5. Conclusions

This work utilized mixture design and response surface design in the formulation development of directly compressible tablets incorporating TSM extract with synergistic antioxidant activity. Mixture design by augmented simplex lattice design was utilized to determine the mixture with optimal synergistic antioxidant activity. It was found that the original TSM formula comprising an equal mass ratio of three *Terminalia* dried fruits exhibited synergistic antioxidant activity. Next, a response surface design using the Box–Behnken method was utilized to optimize the formulation and preparation conditions of the directly compressible tablets of TSM extract. Optimal conditions were found to be a compressional force of 1000 psi, 2% SSG, and 0.5% MgSt. Medium to high dissolution of certain phenolic compounds was observed. Furthermore, the tablets were stable when stored at 30°C/75% RH and 45°C/75% RH for 3 months. In conclusion, the mixture design and response surface design were successfully utilized in the optimization of TSM extract tablets with synergistic antioxidant activity.

## Figures and Tables

**Table 1:** The mass ratio of each plant contained in the TSM herbal formula based on an augmented simplex lattice design.

**Code**	**Mass ratio**		
	**TC**	**TA**	**TB**
TSM 1	1.00	0.00	0.00
TSM 2	0.00	1.00	0.00
TSM 3	0.00	0.00	1.00
TSM 4	0.50	0.50	0.00
TSM 5	0.50	0.00	0.50
TSM 6	0.00	0.50	0.50
TSM 7	0.67	0.17	0.17
TSM 8	0.17	0.67	0.17
TSM 9	0.17	0.17	0.67
TSM 10	0.33	0.33	0.33
TSM 11	0.33	0.33	0.33
TSM 12	0.33	0.33	0.33

**Table 2:** Factors for preparation of directly compressible tablets incorporating TSM extract with synergistic antioxidant activity based on the Box–Behnken design.

**Code**	**Force (psi)**	**SSG (%)**	**MgSt (%)**
TSM-T 1	500	0	1
TSM-T 2	1500	0	1
TSM-T 3	500	4	1
TSM-T 4	1500	4	1
TSM-T 5	500	2	0.5
TSM-T 6	1500	2	0.5
TSM-T 7	500	2	1.5
TSM-T 8	1500	2	1.5
TSM-T 9	1000	0	0.5
TSM-T 10	1000	4	0.5
TSM-T 11	1000	0	1.5
TSM-T 12	1000	4	1.5
TSM-T 13	1000	2	1
TSM-T 14	1000	2	1
TSM-T 15	1000	2	1
TSM-T 16	1000	2	1
TSM-T 17	1000	2	1

**Table 3:** Predicted values, actual values, and error of the prediction of CI value of IC_50_ values obtained from DPPH and FRAP assays.

**Parameters**	**Predicted values**	**Actual values (** **n** = 3**)**	**Error** ^a^ ** (%)**
CI value of IC_50_ values obtained from DPPH assay	0.72	0.67 ± 0.08	−7.46
CI value of IC_50_ values obtained from FRAP assay	0.67	0.68 ± 0.09	1.47

^a^Error = (actual value − predicted value) × 100/actual value.

**Table 4:** ANOVA for the quadratic model of tablet thickness, hardness, friability, and DT of directly compressible tablets incorporating TSM extract with synergistic antioxidant activity and their fit statistics data.

**Source**	**Thickness**		**Hardness**		**Friability**		**DT**	
	**Coefficient**	**p** ** value**	**Coefficient**	**p** ** value**	**Coefficient**	**p** ** value**	**Coefficient**	**p** ** value**
Model	—	< 0.0001^∗^	—	< 0.0001^∗^	—	0.0004^∗^	—	< 0.0001^∗^
Intercept	4.1380	—	6.2600	—	0.6300	—	2.9980	—
*X*_1_-force	−0.505	< 0.0001^∗^	4.0875	< 0.0001^∗^	−1.1688	< 0.0001^∗^	11.2763	< 0.0001^∗^
*X*_2_-SSG	−0.0125	0.0938	−0.0375	0.6295	0.2175	0.0945	−2.0225	0.0063^∗^
*X*_3_-MgSt	0.0025	0.7098	−0.0500	0.5228	0.3163	0.0261^∗^	1.8013	0.0111^∗^
*X*_1_ *X*_2_	0.0075	0.4381	0.2500	0.0490^∗^	−0.2600	0.1463	−2.9125	0.0058^∗^
*X*_1_ *X*_3_	−0.0175	0.0966	0.1750	0.1400	−0.5025	0.0160^∗^	2.3200	0.0169^∗^
*X*_2_ *X*_3_	−0.0025	0.7920	0.0750	0.4987	0.0750	0.6518	−0.0825	0.9148
*X*_1_^2^	0.1648	< 0.0001^∗^	−0.2550	0.0417^∗^	0.9938	0.0004^∗^	8.086	< 0.0001^∗^
*X*_2_^2^	−0.0353	0.0054^∗^	0.4200	0.0046^∗^	−0.2088	0.2203	0.4835	0.5261
*X*_3_^2^	−0.0403	0.0027^∗^	0.2450	0.0481^∗^	−0.0463	0.7742	0.6910	0.3722
Lack of fit	—	0.1432	—	0.7338	—	0.2218	—	0.3417
*R* ^2^	0.9989	—	0.9977	—	0.9614	—	0.9892	—
Adjusted *R*^2^	0.9975	—	0.9948	—	0.9118	—	0.9753	—
Predicted *R*^2^	0.9873	—	0.9882	—	0.5885	—	0.9003	—
Adequate precision	75.0386	—	54.4890	—	14.9465	—	24.8756	—
SD	0.0182	—	0.2103	—	0.3183	—	1.4900	—
Mean	4.1800	—	6.4500	—	0.9776	—	7.3600	—
CV, %	0.4365	—	3.2600	—	32.5600	—	20.2200	—

^*^Significant values (*p* < 0.05).

**Table 5:** Predicted values, actual values, and error of the prediction of tablet thickness, hardness, friability, and DT of TSM extract tablets.

**Parameters**	**Predicted values**	**Actual values**	**Error** ^a^ ** (%)**
Thickness (mm)	4.10	4.12 ± 0.01	0.49
Hardness (kP)	6.55	6.85 ± 0.24	4.38
Friability (%)	0.27	0.30	10.00
DT (min)	1.89	1.81 ± 0.16	−4.42

^a^Error = (actual value − predicted value) × 100/actual value.

## Data Availability

The data used to support the findings of this study are available from the corresponding author upon request.
